# Vertical Gate-All-Around Nanowire GaSb-InAs Core-Shell n-Type Tunnel FETs

**DOI:** 10.1038/s41598-018-36549-z

**Published:** 2019-01-17

**Authors:** T. Vasen, P. Ramvall, A. Afzalian, G. Doornbos, M. Holland, C. Thelander, K. A. Dick, L. - E. Wernersson, M. Passlack

**Affiliations:** 1 TSMC Corporate Research, Kapeldreef 75, 3001 Leuven, Belgium; 2 Lund University, Lund, Sweden

## Abstract

Tunneling Field-Effect Transistors (TFET) are one of the most promising candidates for future low-power CMOS applications including mobile and Internet of Things (IoT) products. A vertical gate-all-around (VGAA) architecture with a core shell (C-S) structure is the leading contender to meet CMOS footprint requirements while simultaneously delivering high current drive for high performance specifications and subthreshold swing below the Boltzmann limit for low power operation. In this work, VGAA nanowire GaSb/InAs C-S TFETs are demonstrated experimentally for the first time with key device properties of subthreshold swing *S* = 40 mV/dec (*V*_*d*_ = 10 mV) and current drive up to 40 μA/wire (*V*_*d*_ = 0.3 V, diameter *d* = 50 nm) while dimensions including core diameter *d*, shell thickness and gate length are scaled towards CMOS requirements. The experimental data in conjunction with TCAD modeling reveal interface trap density requirements to reach industry standard off-current specifications.

## Introduction

Tunneling Field-Effect Transistors (TFETs) are considered a candidate to replace or supplement conventional Complementary Metal Oxide Semiconductor (CMOS) technology^1,2^. TFETs offer the possibility of supply voltage (*V*_*dd*_) scaling and reduced power consumption because the subthreshold swing (*S*) can be less than that of a MOSFET, which is limited to 60 mV/dec at room temperature. In particular, TFETs based on the InAs-GaSb heterostructure are attractive because the broken-band alignment between InAs and GaSb allows for high tunneling transmission and high current drive^3,4^. Recently, there have been experimental demonstrations based on these materials of vertical gate-all-around (VGAA) nanowire (NW) axial InAs/GaAsSb/GaSb TFETs with *S* below 60 mV/dec^5–7^. VGAA NW axial InGaAs/InAs^8^ and VGAA NW axial InAs/Si^9^ TFETs have also displayed *S* below 60 mV/dec.

**Figure 1:**
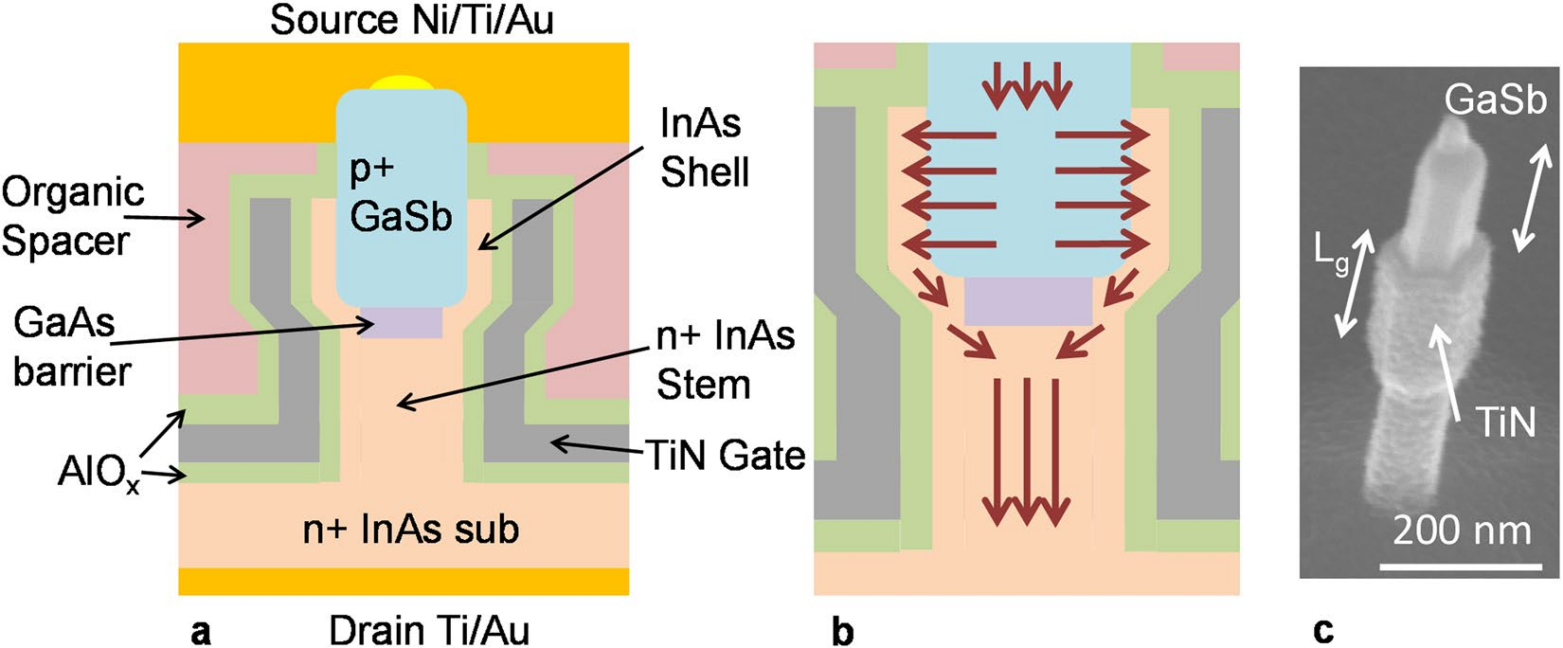
(**a**) Schematic of VGAA C-S TFET (**b**) Magnification of (**a**) with illustration of electron flow in the device (**c**) SEM image of a fabricated device after digital etching of InAs shell in the region of the NW above the gate metal.

In this article, we experimentally demonstrate for the first time VGAA NW GaSb-InAs core-shell (C-S) nTFETs which are predicted to have the highest current-drive capability of any TFET at scaled dimensions and low supply voltage^10^. A device schematic and scanning electron micrograph (SEM) image of the device is shown in Fig. 1. In such a device, a *p*+ GaSb core is wrapped by a thin InAs shell and high-k/metal gate. If properly designed, electrons in the GaSb source tunnel perpendicular to the gate into the InAs shell and are collected by the InAs drain as illustrated in Fig. 1b. The GaAs layer acts as a barrier to suppress axial tunneling. This type of TFET, in which the tunneling direction is parallel to the gate electric field, is sometimes referred to as a “line-tunneling” TFET^11^. There have been several planar embodiments of III-V line TFETs demonstrated in the literature previously^12–14^ though none demonstrate *S* < 60 mV/dec and device dimensions are micron scale. A 50 nm core diameter NW GaSb/InAs C-S TFET was previously reported^15^, however this device was fabricated with a lateral geometry resulting in an ungated shell on the underside on the NW and suffered from high-leakage currents at room temperature and poor *S*.

Here, we demonstrate significant experimental advancement of C-S TFETs and several key properties and physics of C-S TFETs including the narrowest device dimensions reported with GaSb core of 35 nm, *S* down to 40 mV/dec at *V*_*d*_ = 10 mV, high drive current up to 40 μA/wire at *V*_*d*_ = 0.3 V and *d* = 50 nm, and negative differential resistance (NDR) when the GaSb/InAs junction is forward biased, which confirms band-to-band tunneling (BTBT) transport is present in the device. We show that current flow in the device is indeed through the InAs shell by comparison with reference devices fabricated from identical NWs grown intentionally without shell. We also determine that measured off current and *S* are limited by trap-assisted tunneling due to interface traps (*D*_*it*_) at the high-k/InAs interface. Using temperature-dependent *I-V* and TCAD modeling, it is found that tunneling into traps within the band gap of InAs at the high-k/InAs interface and subsequent thermal emission into the InAs conduction band limits *S* to typically 110 mV/dec or greater at *V*_*d*_ = 0.3 V. We then calculate the required *D*_*it*_ limits in order to achieve industry standard off-current requirements for CMOS technology. Finally, the impact of GaSb core doping is shown and found to be a critical parameter necessary for high drive current.

## Results and Discussion

**Figure 2:**
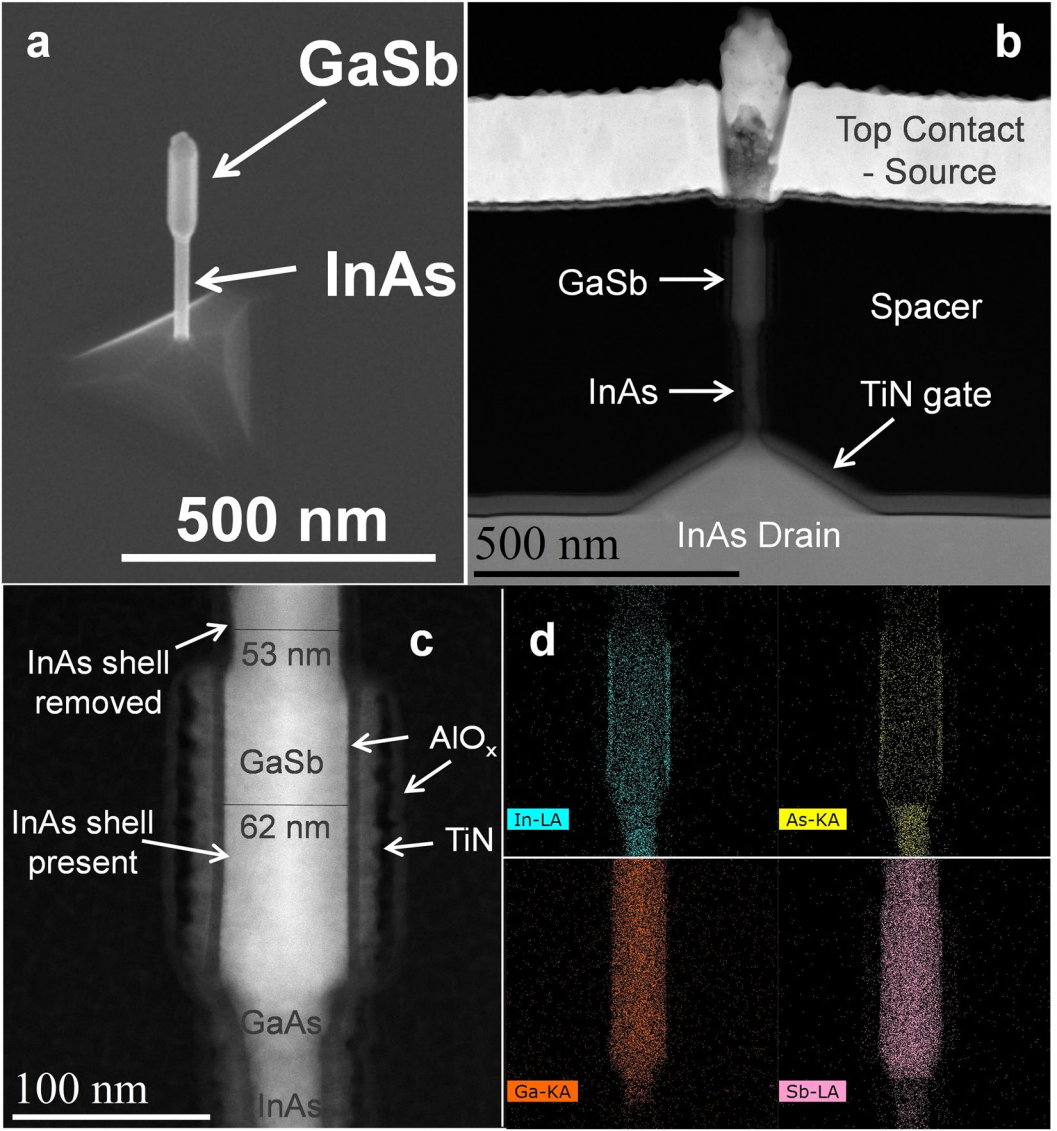
(**a**) SEM image of an as-grown InAs stem, GaSb core, InAs shell nanowire. (**b**) and (**c**) HAADF STEM image of a fabricated VGAA NW GaSb-InAs C-S TFET with GaSb core diameter of 53 nm and InAs shell of 4.5 nm (**d**) EDX maps of Ga (orange), As (yellow), In (light blue), and Sb (purple). In (**c**) it is evident the InAs shell is etched in the region above the gate metal by the reduction of the NW diameter. In the EDX spectra, the brighter In and As signals at the edges of the NW also reveal the presence of a shell. The GaAs segment is also evident and is approximately 24 nm long.

C-S GaSb/InAs NWs were grown by metal organic chemical vapor deposition (MOCVD) using the vapor liquid solid (VLS) method from Au particle catalysts in predefined locations on an InAs substrate. Figure 2a shows a SEM image of a NW following MOCVD growth. A 300–400 nm long Sn-doped *n*-type InAs segment is first grown, followed by approximately a 20 nm long undoped GaAs segment, and 300–400 nm long Zn-doped *p*-type GaSb segment. The diameter of the InAs stem ranges from 23–32 nm and the GaSb core diameter ranges from 35–60 nm. Subsequently, an undoped InAs shell of approximately 3–5 nm is grown over the entire structure. To our knowledge, these are the narrowest C-S TFET structures reported. Even further diameter scaling of the nanowires towards CMOS relevant dimensions is possible and demonstrated for nanowires grown with Au colloids. The supplementary information includes grown GaSb-InAs C-S nanowires with GaSb core diameter of 17 nm and InAs shell of 3 nm. Further description of the growth process is outlined in the methods section. VGAA NW GaSb-InAs C-S TFET devices, as shown in Fig. 1, were fabricated using the process flow also described in the methods section below. Further detail of the device structure is shown in Fig. 2b–d, high-angle annular dark-field scanning transmission electron microscopy (HAADF STEM) images of a fabricated device and energy-dispersive x-ray spectroscopy (EDX) mapping of the constituent elements of the NW. The EDX spectra reveal the presence of an InAs shell and GaAs barrier layer. The images also confirm the InAs shell is removed in the region above the gate metal and is approximately 4.5 nm or less.

**Figure 3:**
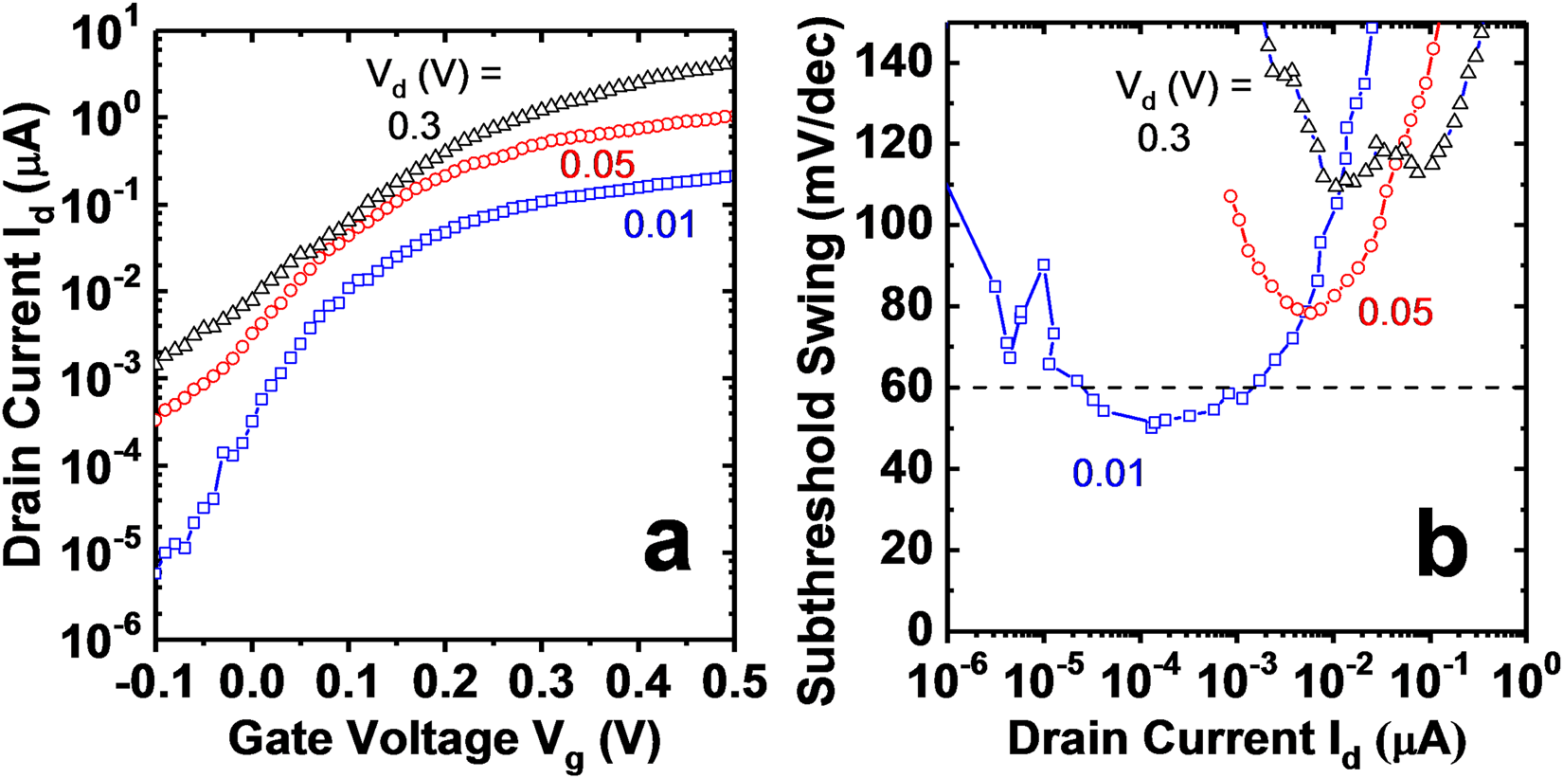
(**a**) *I*_*d*_ − *V*_*g*_, (**b**) *S* − *I*_*d*_, for a C-S TFET device with GaSb diameter of 35 nm, InAs shell thickness approximately 4 nm, and *L*_*g*_ approximately 35 nm. *S*_*min*_ equals 50 mV/dec at *V*_*d*_ = 10 mV.

**Figure 4:**
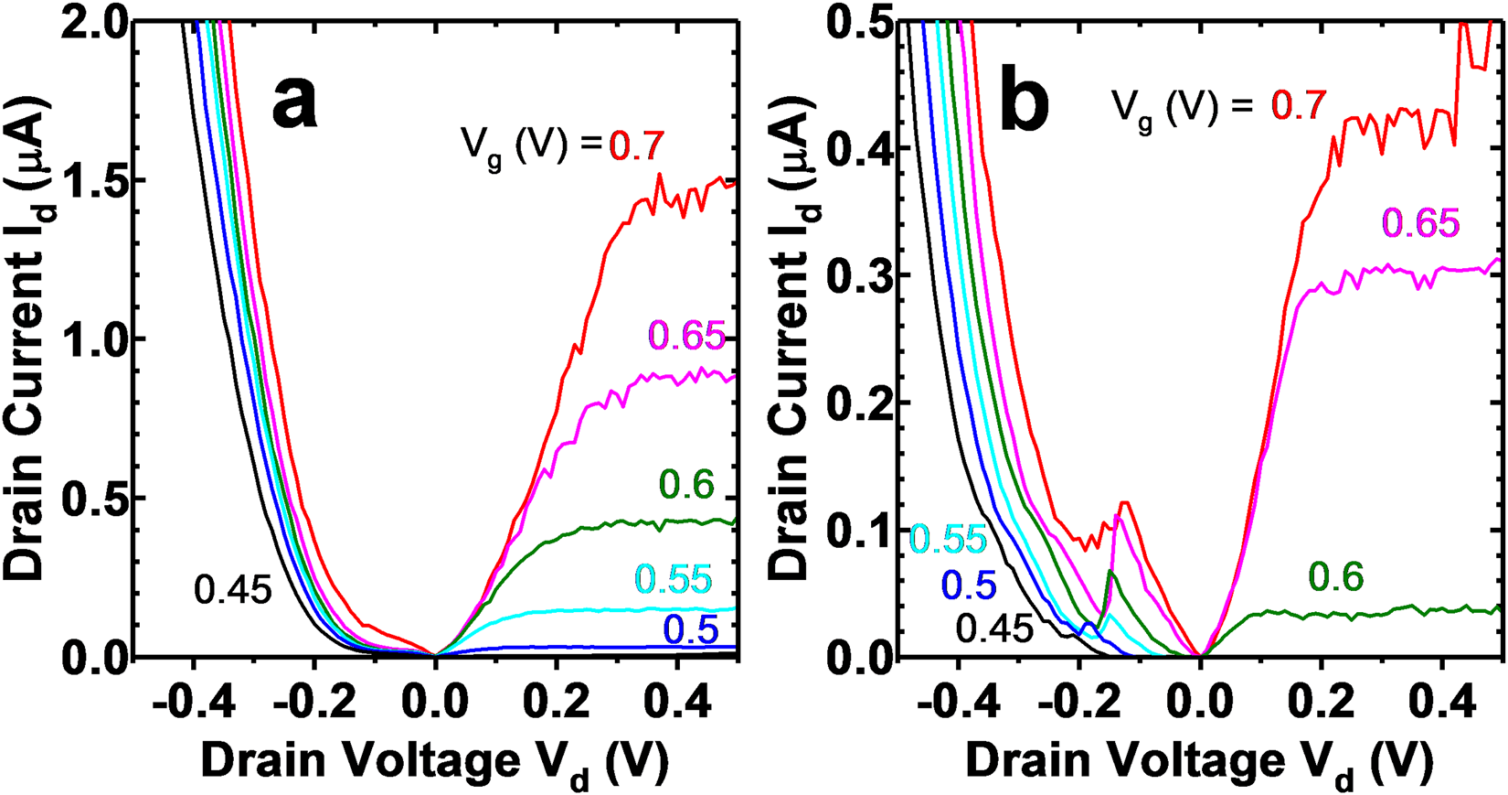
(**a**) *I*_*d*_ − *V*_*d*_ at 300 K and (**b**) 10 K for a C-S TFET. Negative differential resistance is visible for negative *V*_*d*_ at 10 K, an indication of band-to-band-tunneling operation in the device. The noise present in the saturation region may be due to discrete trapping events in the high-k and is commonly observed in nanowire FET devices of small dimensions^8^.

Figure 3 shows *I*_*d*_ − *V*_*g*_ and *S* − *I*_*d*_ of a single NW device with GaSb core diameter approximately 35 nm. At *V*_*d*_ = 10 mV, minimum subthreshold swing equals 50 mV/dec, below the thermal limit for a MOSFET at room temperature. *S* is less than 60 mV/dec for approximately 2 orders of magnitude in drain current with *I*_60_ = 1.5 nA/wire. *I*_60_ is defined as the current at which *S* increases above 60 mV/dec, and is desired to be as high as possible in order for the steep subthreshold swing to be technologically relevant. As *V*_*d*_ increases from 10 mV to 300 mV, the off-state current increases to 1.4 nA/wire and *S* increases to 110 mV/dec. The *V*_*d*_ dependence of *S* requires further experimental verification with additional structural modifications, which was not studied in this work. A possible mechanism for the increase of *S* is drain leakage due to the overlapped gate and drain. This could be studied by the addition of a bottom spacer in the device. The current drive at *V*_*d*_ = 0.3 V, *V*_*g*_ = 0.5 V is 4.3 μA/wire or 39.4 μA/μm normalized to GaSb core diameter of 35 nm. For the majority of fabricated devices, including the above, gate leakage is at the measurement noise floor. In Fig. 4, the output characteristics show asymmetry with drain voltage, though NDR is not observed at room temperature. However, reduction of the temperature to 10 K reveals the presence of NDR, confirming tunneling operation in the device.

**Figure 5:**
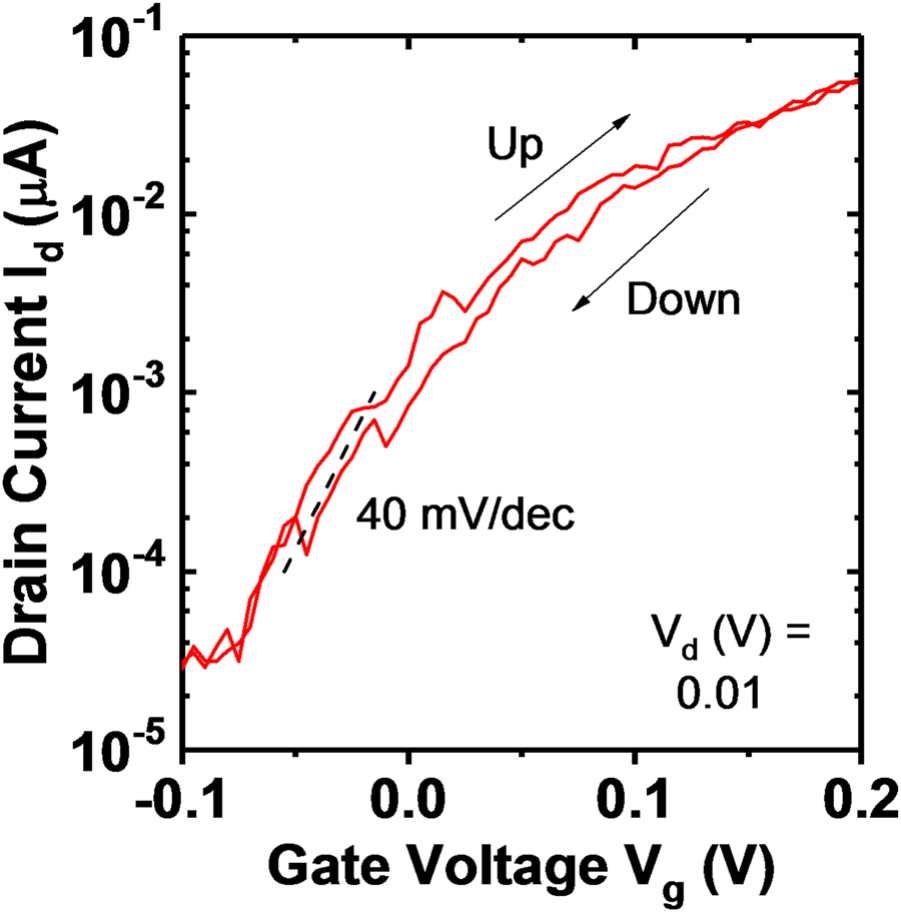
*I*_*d*_ − *V*_*g*_ at *V*_*d*_ = 10 mV for a different device with the steepest measured subthreshold swing of approximately 40 mV/dec. Hysteresis is also shown and is approximately 10 mV where *S* = 40 mV/dec. The curve is swept from down to up. Distinct steps are observed which may be attributed to oxide defects as shown for axial TFETs^16^. The minimum average swing over one decade in current is degraded from 42 to 56 mV/dec due to the presence of a step.

Further devices measured also display *S* < 60 mV/dec at *V*_*d*_ = 10 mV. The steepest *S* measured was found to be 40 mV/dec at *V*_*d*_ = 10 mV. This is the steepest reported *S* for a core-shell TFET architecture. Figure 5 shows the *I*_*d*_ − *V*_*g*_ including hysteresis. Distinct steps are observed in the current, particularly for the down sweep. Similar steps were observed in axial TFETs^16^ and were attributed to oxide defects. The minimum subthreshold swing in between the steps is approximately 40 mV/dec, however the steps degrade the average swing over one decade in current from 42 to 56 mV/dec.

### Core-Shell TFET On Performance

**Figure 6:**
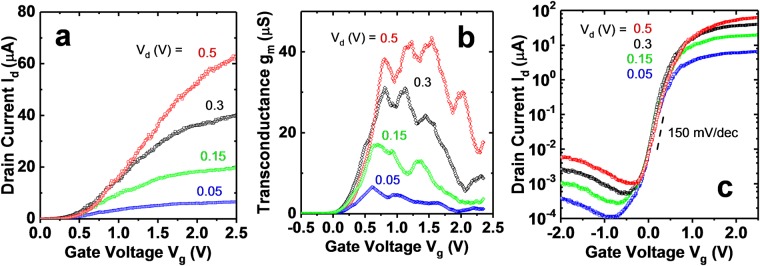
(**a**) *I*_*d*_ − *V*_*g*_ (linear scale) for *V*_*d*_ = 0.5, 0.3, 0.15, and 0.05 V for a device with the highest measured current drive up to 40 μA/wire and 60 μA/wire for *V*_*d*_ = 0.3 and 0.5 V, respectively. The device has a GaSb core diameter of approximately 50 nm. (**b**) *g*_*m*_ − *V*_*g*_, and (**c**) *I*_*d*_ − *V*_*g*_ (log scale), with *S*_*min*_ = 150 mV/dec. The multiple peaks in *g*_*m*_ are likely due to multiple bound states in the InAs shell, similar to the devices from Dey^15^. A thorough analysis of this is beyond the scope of this manuscript.

C-S TFETs have been thought to be attractive due to the line tunneling nature of the device, which should provide enhanced current drive. On current up to 27 μA/wire at *V*_*d*_ = 0.3 V and *I*_*off*_ = 1 pA/wire is predicted by atomistic simulation for an optimized GaSb-InAs C-S TFET with GaSb diameter of 6.6 nm^10^. Experimentally, the device with highest measured drive current and transconductance is shown in Fig. 6. In this case, drive current up to 40 μA/wire at *V*_*d*_ = 0.3 V for a device with 50 nm GaSb core diameter is measured for *V*_*g*_ = 2.5 V. The large gate overdrive necessary is consistent with having high *D*_*it*_ (to be discussed later) and a large effective oxide thickness (EOT) of 3.5 nm. The EOT is calculated assuming an AlO_x_ dielectric constant of 9 and thickness of 8 nm. In addition, there is no engineered alignment in energy of the InAs shell conduction band over the GaSb with respect to that over the GaAs barrier, as predicted to be necessary by atomistic simulation for a steep turn on of line tunneling^10^. This will add to the necessary gate overdrive. Comparing to the predicted value from atomistic simulation^10^, the measured current drive is approximately a factor of 5–38x less, dependent on an assumption of current to either scale linearly with the GaSb diameter or with the square of the GaSb diameter. The discrepancy is most likely due to insufficient GaSb doping (to be discussed later). The GaSb doping concentration used in the atomistic simulations is 1 × 10^20^ cm^−3^. For comparison with previously demonstrated C-S NW GaSb-InAs TFETs, Dey^15^ reported a maximum current of approximately 8 μA/wire at *V*_*d*_ = 0.3 V, *V*_*g*_ = 1.5 V for a similar GaSb core diameter. Note that this device also turned off poorly, with the minimum measured current approximately 0.2 μA at *V*_*d*_ = 0.3 V for *V*_*g*_ = −1.3 V. Comparing to the InAs/GaAsSb/GaSb axial TFET^5^ with GaSb diameter of approximately 70 nm, the maximum current reported is approximately 4 μA/wire at *V*_*d*_ = 0.3 V for *V*_*g*_ = 0.5 V. Thus, the present devices demonstrate for the first time the current-drive capability of the core-shell architecture, although further improvements or changes to the device (discussed later) are necessary to simultaneously achieve the high current drive with steep *S*.

### Verification of Radial Core-Shell Transport

**Figure 7:**
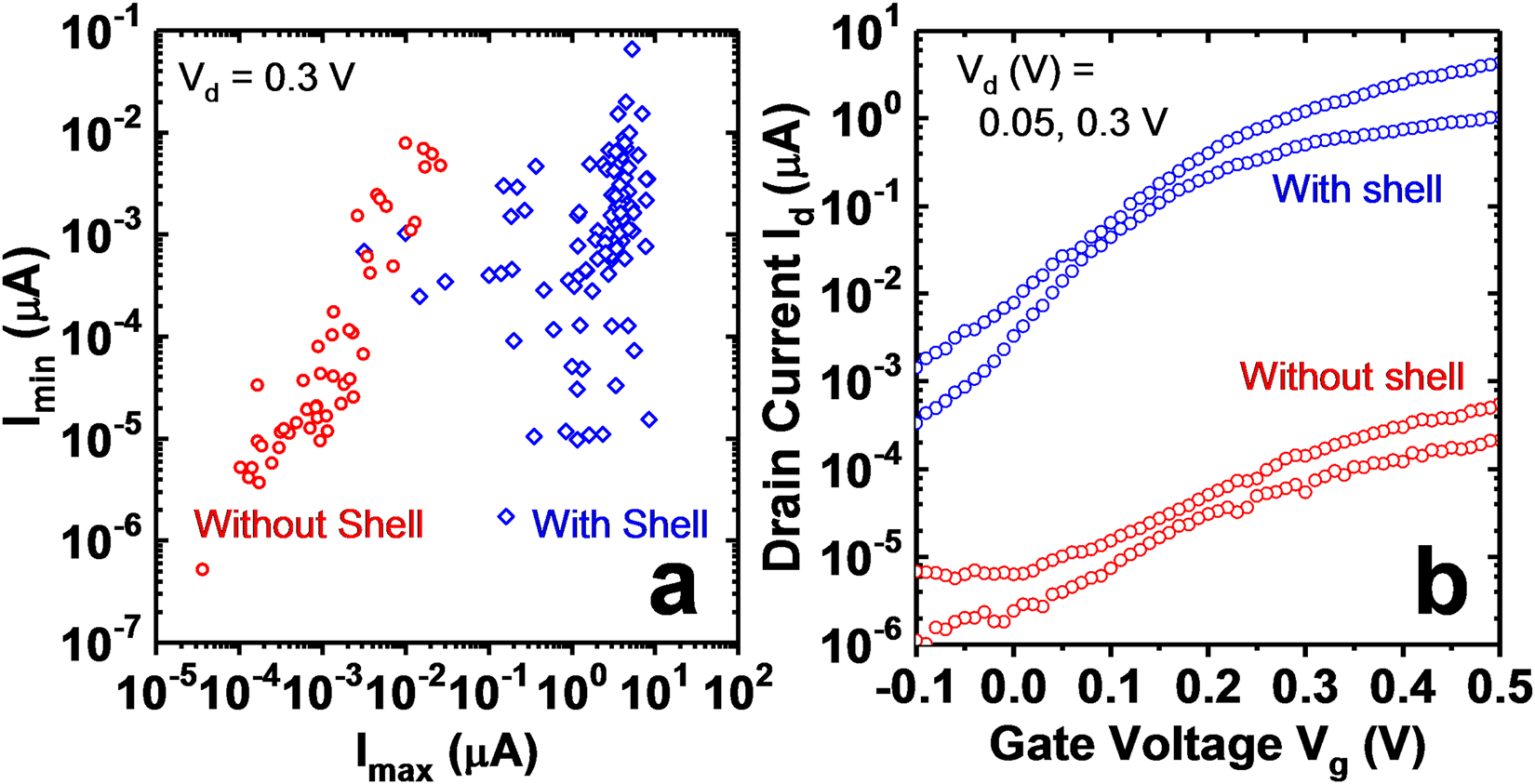
(**a**) *I*_*min*_ versus *I*_*max*_ at *V*_*d*_ = 0.3 V for devices grown intentionally without InAs shell (red circles) and devices with shell grown for 3 min (blue diamonds). Each point has the same gate-voltage sweep range. (**b**) *I*_*d*_ − *V*_*g*_ at *V*_*d*_ = 0.05 and 0.3 V for a device with shell (blue circle) and grown intentionally without shell (red circle). The devices without shell provide a measure of the axial leakage across the GaAs barrier. Axial leakage is at least 100× less than the measured current.

In order to verify if the observed current was flowing through the InAs shell rather than axially across the GaAs barrier, reference devices were fabricated which were grown intentionally without an InAs shell. These devices provide a direct measure of the axial leakage across the GaAs barrier. Reference devices were fabricated with the same process flow, with appropriate modifications to account for the lack of InAs shell. Fig. 7a shows a plot of *I*_*max*_ versus *I*_*min*_ at *V*_*d*_ = 0.3 V for a population of devices intentionally grown without shell and another with shell grown for 3 min. *I*_*max*_ and *I*_*min*_ are defined as the maximum and minimum measured current within the measured gate-voltage range. Each point is measured with the same gate-voltage range. Clearly, there is a difference of greater than 100 × in current levels for the two populations. This confirms transport is occurring within the shell in the C-S TFET devices. Fig. 7b shows example *I*_*d*_ − *V*_*g*_ curves for a device from each.

### Off Current and Subthreshold Swing Limits

**Figure 8:**
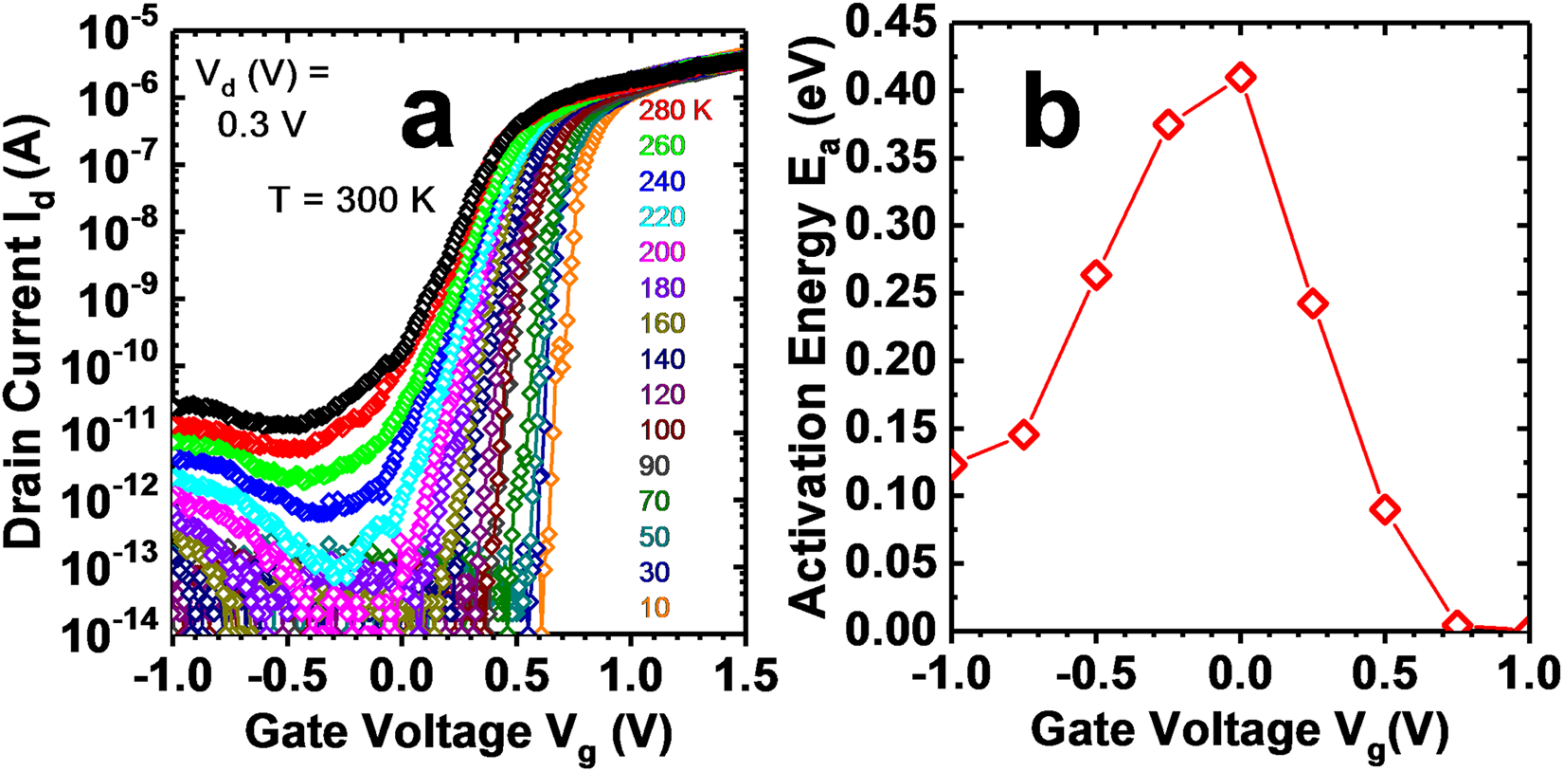
(**a**) *I*_*d*_ − *V*_*g*_ versus temperature at *V*_*d*_ = 0.3 V for a C-S TFET device. Off current and subthreshold swing both reduce with reducing temperature. (**b**) Activation energy extracted from an Arrhenius plot of data in (**a**) versus gate voltage.

To further investigate the off-current and subthreshold swing limitations, temperature dependent measurements were performed from room temperature down to 10 K. Fig. 8 shows temperature dependent *I*_*d*_ − *V*_*g*_ at *V*_*d*_ = 0.3 V for a C-S TFET device. There is a large temperature dependence, particularly in the off and subthreshold regime. The minimum current reduces to the noise floor near 200 K, and the minimum subthreshold swing reduces with temperature down to 15 mV/dec at 10 K. The gate leakage (not shown) is at the noise floor for all measurements. An activation energy, *E*_*a*_, was also extracted by constructing an Arrhenius plot of the natural log of the current versus 1/*kT*. Fig. 8b shows *E*_*a*_ versus gate voltage. *E*_*a*_ has a peak value of 0.41 eV at zero gate bias and reduces as the gate bias increases to nearly 0 eV in the on state. In the on state, the low activation energy is an indication of band-to-band tunneling^17^. *E*_*a*_ also reduces for more negative gate voltage in the region where the current starts increasing with additional negative gate bias. This might be explained by ambipolar tunneling current in the InAs drain.

**Figure 9:**
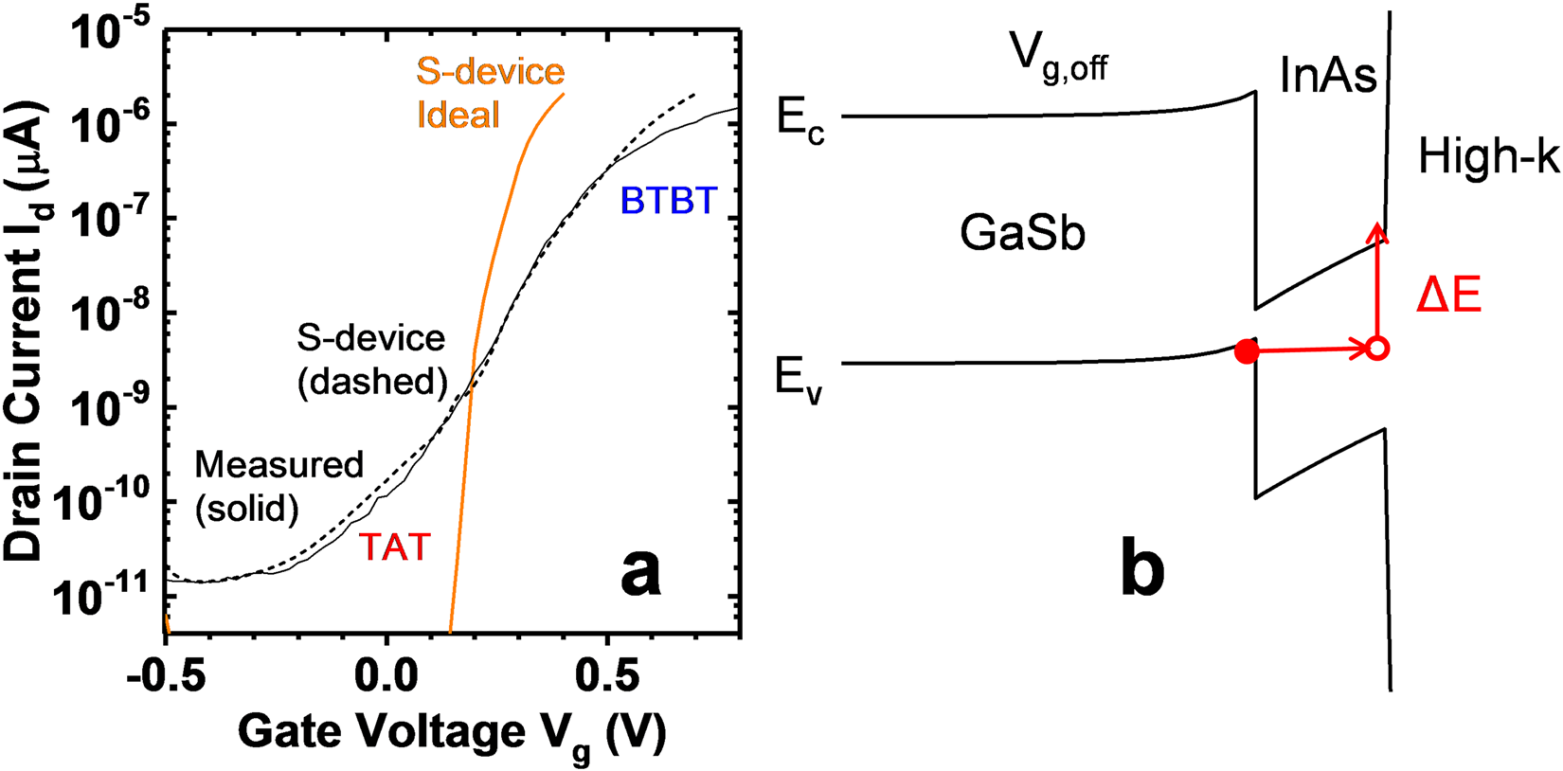
(**a**) Measured (solid black), simulated ideal (solid orange) and simulated with traps (dashed black) *I*_*d*_ − *V*_*g*_ at *V*_*d*_ = 0.3 V. For the dashed curve the trap parameters use *D*_*it*_ = 1.4 × 10^13^ cm^−2^eV^−1^ and σ = 10^−14^ cm^2^. (**b**) Energy band diagram perpendicular to the gate in the channel region for a gate bias in the off state. The trap assisted tunneling and thermal emission process is illustrated in red.

To further understand the physical mechanisms for the observed temperature dependence, Sentaurus Device^18^ (s-device) was used to model the device structure. The details of the simulation can be found in the supporting information. A Gaussian *D*_*it*_ distribution with peak at 1.4 × 10^13^ cm^−2^eV^−1^ centered at the InAs conduction band edge (*E*_*c*_) and at the high-k/InAs interface was included to account for defects in the device. This *D*_*it*_ distribution is similar to experimentally obtained values for InAs with similar surface treatment prior to high-k deposition and high-k^19^. Other defects, such as traps at GaSb-InAs, GaSb-GaAs, or GaAs-InAs interface were not included as none were observed in TEM. In addition, NWs are known to accommodate lattice mismatch without generation of defects^20^. Fig. 9 shows *I*_*d*_ − *V*_*g*_ of the simulated device, both ideal and with traps included compared to the measured curve, and an energy band diagram to aid in understanding the trapping mechanism. When comparing the ideal with the measured curve, it is obvious the effect of traps is significant and must be included to predict the measured current. The device with traps has a degraded subthreshold swing and a plateau in the off state. The *D*_*it*_ contributes an electrostatic degradation of the subthreshold swing, but the more dominant degradation is the trap-assisted tunneling current generated due to the presence of traps. The mechanism of trapping is understood with help from the energy band diagram in b), a cut perpendicular to the gate in the channel region. Electrons in the valence band of GaSb tunnel into empty trap states in the energy gap of InAs at the high-k/InAs interface and subsequently are thermally emitted into the conduction band. This process gives rise to a trap-assisted tunneling current. It is worth noting that this TAT process should be NW core diameter independent for the same InAs shell and high-k thickness. This is consistent with similar measured *S* values over a large number of devices of approximately 110–150 mV/dec at *V*_*d*_ = 0.3 V for both 35 and 50 nm GaSb core diameter devices.

It is now apparent that to improve subthreshold swing and reduce off current in experimental devices, mitigation or reduction of *D*_*it*_ is critical in order to suppress trap-assisted tunneling current. To estimate the required *D*_*it*_ necessary to reduce TAT current and reach specified off-current levels, the magnitude of *D*_*it*_ was varied in s-device simulations. To reach an off-current requirement of 100 pA/wire, a *D*_*it*_ ≤ 10^12^ cm^−2^eV^−1^ is calculated. *D*_*it*_ ≤ 10^12^ cm^−2^eV^−1^ has been previously reported for example, by using an oxygen-terminated InAs surface^21^. To reach an off-current requirement of 1 pA/wire, a *D*_*it*_ ≤ 10^10^ cm^−2^eV^−1^ is calculated. For reference, the 2017 International Roadmap for Devices and Systems (IRDS) predicts a low power off-current requirement of 2.4 pA/wire in the year 2030 for a VGAA architecture^22^. Reaching the IRDS target likely would require alternative methods yet to be demonstrated to mitigate the traps.

### GaSb Doping Effect

**Figure 10:**
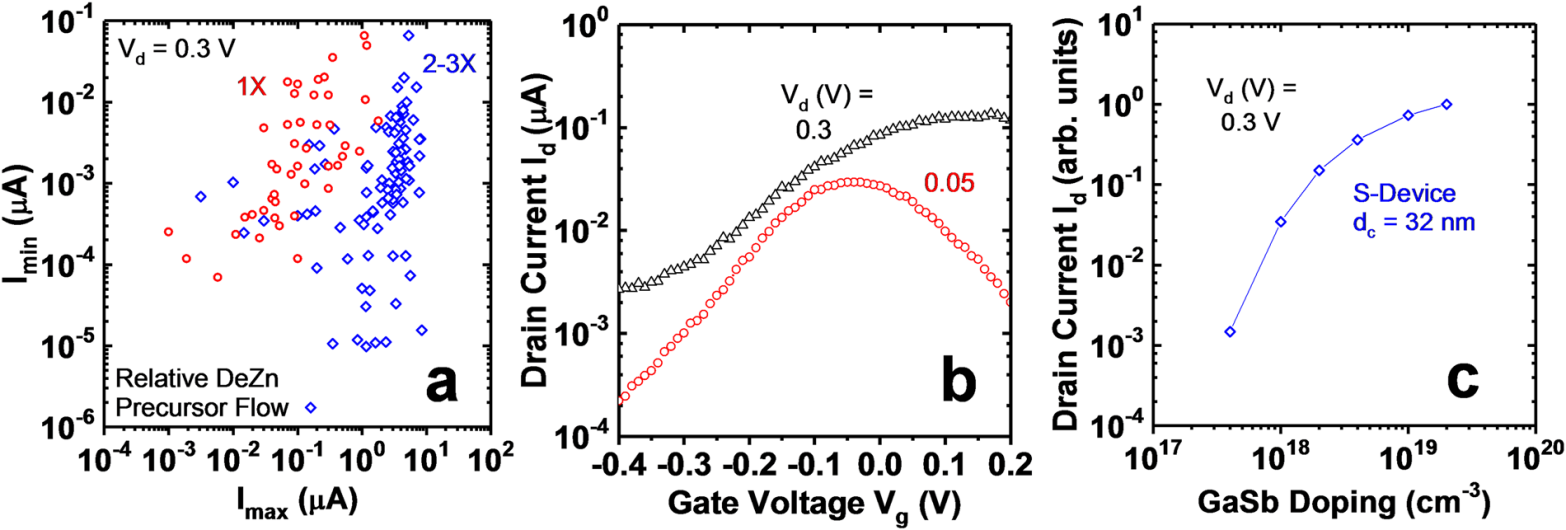
(**a**) *I*_*min*_ versus *I*_*max*_ at *V*_*d*_ = 0.3 V for different GaSb Zn doping. The doping is controlled by the diethylzinc (DEZn) precursor flow during growth. The blue diamonds have 2–3x flow relative to the red diamonds and typically ~40x maximum current. Each point has the same gate voltage sweep range. (**b**) An example *I*_*d*_ − *V*_*g*_ for a 1x GaSb doped device at 0.3 V (black triangle) and 0.05 V (red circle). The reduction of current with increasing gate voltage at *V*_*d*_ = 0.05 V is a sign of source depletion and insufficient GaSb doping. (**c**) Relative on current versus GaSb doping concentration for a given GaSb core diameter *d*_*c*_ = 32 nm as predicted by s-device. This illustrates the importance of having as high as possible GaSb doping concentration.

Finally, the effect of GaSb doping on device performance was investigated. GaSb doping is controlled by the diethylzinc (DEZn) precursor flow during growth of the NWs. Fig. 10a shows *I*_*max*_ versus *I*_*min*_ at *V*_*d*_ = 0.3 V for a population of devices with different relative DEZn precursor flows. Devices with 2× or higher DEZn flow exhibit approximately 40× higher maximum current levels than those with 1× flow. In addition, devices with 1× flow exhibit source depletion effects in the *I*_*d*_ − *V*_*g*_ curve as seen in Fig. 10b. Most pronounced at low drain bias (due to the additional effect of saturation of the energy windows available for tunneling that is more pronounced at low drain bias^23^), the current peaks and then reduces as gate bias increases. This occurs because the gate bias depletes the GaSb due to insufficient doping, which increases the barrier length for tunneling. GaSb doping is thus a critical parameter in a C-S TFET device and must be sufficiently high to obtain high current levels. Fig. 10c further illustrates the importance of having sufficiently high GaSb doping. Relative on current versus GaSb doping concentration was simulated with s-device. On current reduces by orders of magnitude if the doping is not sufficient. The doping requirements also depend on GaSb core diameter and are higher as the diameter is reduced.

## Conclusions

GaSb-InAs C-S TFETs are promising candidates for a low-voltage CMOS technology due to their high current drive capability. We have advanced the experimental state and demonstrated the first VGAA NW GaSb-InAs C-S TFETs and have shown key properties and physics with *S* down to 40 mV/dec at *V*_*d*_ = 10 mV and room temperature, drive current capability up to 40 μA/wire at *V*_*d*_ = 0.3 V, NDR, transport through the InAs shell, and GaSb core doping effects. *S* and off current were shown to be limited by trap assisted tunneling due to *D*_*it*_ at the high-k/InAs interface. Further improvement of devices requires *D*_*it*_ ≤ 10^12^ cm^−2^eV^−1^ and higher GaSb core doping.

## Methods

### MOCVD Growth of Core-Shell Nanowires

NWs are grown in VLS mode from Au particle catalysts. In VLS growth, a vapor-phase precursor catalytically decomposes at a metal nanoparticle surface, forming a supersaturated eutectic liquid. The solid crystalline nanowire grows by precipitation from the liquid catalyst particle. To form the Au particle catalysts, e-beam lithography was used to pattern holes in resist of 15 or 20 nm in diameter on an InAs 111B substrate with *n*-type sulphur doping = 1 × 10^18^ cm^−3^. 15 nm of Au was e-beam evaporated and standard lift-off was used to define Au particles in pre-defined positions. NW growth took place in an Aixtron CCS closed coupled showerhead MOCVD reactor. Purified hydrogen with a total flow of 8 l/min was used as carrier gas. The precursors used for growth of the NWs were trimethylindium (TMIn), arsine (AsH_3_), and tetraethyltin (TESn) for InAs and trimethylgallium (TMGa), trimethylantimony (TMSb), and diethylzinc (DEZn) for GaSb. The V/III ratio during growth of GaSb was controlled by an Epison feedback controller. The reactor temperature was calibrated by means of a LayTec EpiR TT *in-situ* metrology system. The epitaxy started with a thermal annealing step at 500 °C. Meanwhile the substrate was stabilized by an AsH_3_ flow of 20 ml/min at 100 mbar reactor pressure. After annealing, the susceptor temperature was set to the InAs growth temperature of 460 °C. When the correct growth temperature was attained and stabilized for about 3 minutes InAs NW growth commenced. The InAs(Sn) NW was grown for about 3 minutes then growth was stopped and the reactor temperature was raised to slightly above 500 °C in an AsH_3_ flow. When the reactor temperature was stable AsH_3_ was switched to TMSb followed by the addition of TMGa and DEZn. During this sequence of switching, the growth interrupt time between AsH_3_ turn off and TMGa turn on controls the degree to which a GaAs segment forms due to residual AsH_3_ in the growth chamber. Since a GaAs segment is desired in the structure, the interrupt time was kept short. The GaSb(Zn) segment was grown for 15–20 minutes. Finally, the temperature was lowered and an undoped InAs shell was grown for 3–4 minutes.

### Core-Shell TFET Fabrication

A high-k dielectric layer of AlO_x_ of 5–8 nm is deposited onto the sample by standard atomic layer deposition (ALD) at 150 °C with TMA and H_2_O precursors. Next, 35 nm of TiN is deposited by sputtering to form the gate metal. A planarizing photoresist is spun onto the sample and subsequently thinned by O_2_ plasma to a thickness which reveals the tops of the NWs. The TiN is wet etched from the revealed portion of the NW by H_2_O_2_ at 60 °C and the photoresist removed. Next, the TiN gate pads are formed by standard lithography and an SF_6_ based dry etch. Next, the high-k is wet etched by a 1:800 HF:H_2_O solution. The InAs shell is then wet etched using a digital etch process which consists of O_2_ plasma for 5 min followed by etching in Citric Acid: IPA mixture for 30 s. If the GaSb is exposed, all rinsing steps are performed in isopropanol (IPA) to avoid GaSb exposure to water. Next, 5 nm of AlO_x_ is deposited by ALD as a protective cap of the revealed GaSb segment. A planarizing resist is spun onto the sample, patterned to open vias to the gate and substrate, and hard baked at 200 °C for 40 minutes and thinned by O_2_ plasma to a thickness approximately 50 nm above the TiN gate edge. This resist acts as the top spacer between the gate and top contact. Next, the protective AlOx layer is etched in 1:800 HF:H_2_O solution followed by top contact deposition of 10 nm Ni, 5 nm Ti, and 200 nm Au. Finally, the top contact pads are patterned and the top contact metals are wet etched using a commercially available Au etchant to etch Au, 1:10 HF to etch Ti, and a Ni etchant mixture of 1 H_2_SO_4_: 5 CH_3_COOH: 5 HNO_3_: 15 H_2_O. For fabrication of reference devices without InAs shell, the process is similar, with the exception that the high-k is only removed from the contacted region of the GaSb just prior to source contact deposition.

## Electronic supplementary material

Supporting Information

## Data Availability

Data generated or analyzed during this study are available from the corresponding author upon reasonable request.
