# Supplementary material for: Vertical Gate-All-Around Nanowire GaSb-InAs Core-Shell n-Type Tunnel FETs
Source: Sci Rep. 2019 Jan 17;9:202. doi: 10.1038/s41598-018-36549-z (PMC6336843; doi:10.1038/s41598-018-36549-z)
Supplement: Supplementary file 1 — Supporting Information [file 41598_2018_36549_MOESM1_ESM.pdf]

Supporting Information for:

Vertical Gate-All-Around Nanowire GaSb-InAs Core-Shell n-Type Tunnel FETs

T. Vasen, P. Ramvall, A. Afzalian, G. Doornbos, M. Holland, C. Thelander[1], K.A. Dick[1], L.-E. Wernersson[1], and M. Passlack

TSMC, Kapeldreef 75, 3001 Leuven, Belgium; [1]Lund University, Lund, Sweden

CONTENTS

1. Record narrow grown Core-Shell GaSb-InAs Nanowires
2. Sentaurus Device (s-device) simulation setup

1. Record Narrow Core-Shell GaSb-InAs Nanowires

Figure S1 shows EDX maps and an HRTEM image of a GaSb-InAs core-shell nanowire with GaSb diameter of 17 nm and InAs shell of 3 nm. This is the narrowest GaSb-InAs core-shell nanowire ever reported. The nanowire was grown with similar growth process as described in the manuscript but using randomly placed Au colloids instead of EBL defined

Au particle. Further process improvements will allow to grow such small diameter structures in EBL defined patters.

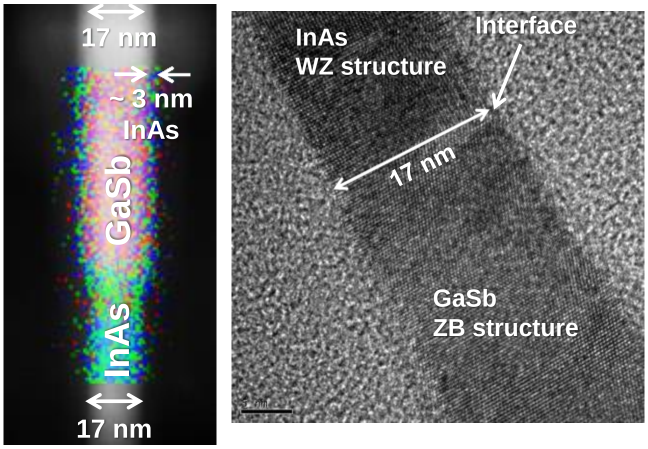

Figure S1. (Left) EDX maps of record narrow GaSb-InAs Core-Shell Nanowire. The GaSb core diameter is 17 nm with an InAs shell of 3 nm. The elemental colors are: In (blue), As (green), Ga (red). Sb is omitted for clarity. (Right) HR TEM of the same nanowire.

2. Sentaurus Device Simulation Setup

Sentaurus Device was used to understand the effect and location of defects on the experimental VGAA NW GaSb-InAs C-S TFET. S-device is well suited for this type of analysis as physical effects can be isolated by model selection. We do not use s-device to predict intrinsic performance for example, as this requires self-consistent quantum transport. Figure S2 shows the GaSb-InAs core-shell TFET structure simulated in s-device. The device dimensions and doping concentrations are shown in the figure. The simulations were performed in cylindrical coordinates. The energy band gap of the InAs shell and the conduction and valence band offsets between the InAs shell and the GaSb core were taken from atomistic simulation which ignored

the small lattice mismatch between InAs and GaSb.[10] The dynamic nonlocal path band-to-band tunneling model was included, with material-dependent tunneling masses. This approach provides a reasonable description of the on-performance, however will always remain qualitative at best due to the lack of coupling between bias-dependent confined band structure and tunneling parameters. The leakage related to traps at the InAs/high-k interface is modeled by explicitly calculating the balance between the capture and emission rates; The capture and emission processes are both local (coupling the interface traps with the InAs valence and conduction bands by thermal processes) and non-local (coupling the interface traps with the GaSb valence band by elastic and phonon-assisted inelastic tunneling) in nature.

The assumed interface trap density D_{it} profile is Gaussian with peak of 1.4 x 10^{13} $cm^{-2}eV^{-1}$ at the InAs conduction band edge at the InAs/high-k interface. The capture cross section is assumed to be 10^{-14} cm^2.

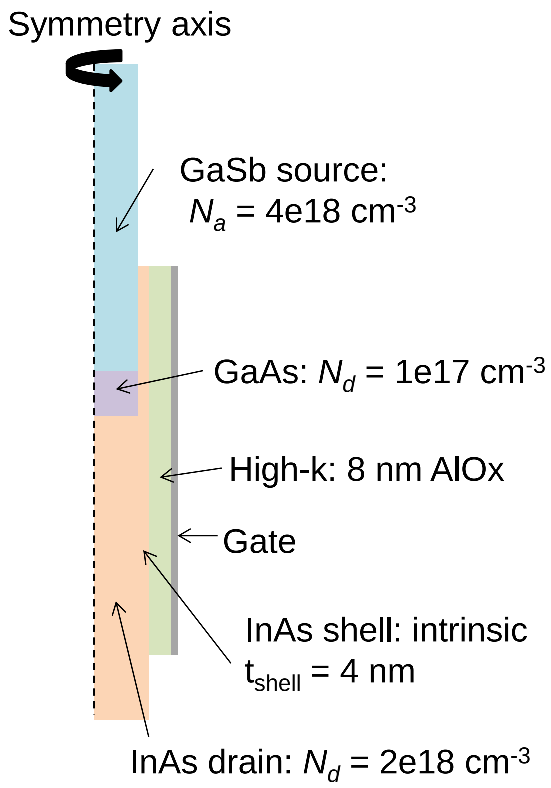

Figure S2. Device structure (to scale) of the GaSb-InAs core shell TFET simulated with Sentaurus Device. The structure is simulated in cylindrical coordinates with symmetry axis on the left. The GaSb core diameter is 32 nm.
